# An Asiatic Chironomid in Brazil: morphology, DNA barcode and bionomics

**DOI:** 10.3897/zookeys.514.9925

**Published:** 2015-07-27

**Authors:** Gizelle Amora, Neusa Hamada, Lívia Maria Fusari, Vanderly Andrade-Souza

**Affiliations:** 1 Instituto Nacional de Pesquisas da Amazônia (INPA), Coordenação de Biodiversidade - CBio, Laboratório de Citotaxonomia e Insetos Aquáticos, Manaus, AM, Brazil; 2 Universidade de São Paulo, Museu de Zoologia (MZUSP), Laboratório de Diptera, São Paulo, SP, Brazil

**Keywords:** Aquatic insects, non-native species, Amazonas, *Chironomus kiiensis*, *Chironomus striatipennis*, sibling species

## Abstract

In most freshwater ecosystems, aquatic insects are dominant in terms of diversity; however, there is a disproportionately low number of records of alien species when compared to other freshwater organisms. The Chironomidae is one aquatic insect family that includes some examples of alien species around the world. During a study on aquatic insects in Amazonas state (Brazil), we collected specimens of Chironomidae that are similar, at the morphological level, to *Chironomus kiiensis* Tokunaga and *Chironomus striatipennis* Kieffer, both with distributions restricted to Asia. The objectives of this study were to provide morphological information on this *Chironomus* population, to investigate its identity using DNA barcoding and, to provide bionomic information about this species. *Chironomus* DNA barcode data were obtained from GenBank and Barcode of Life Data Systems (BOLD) and, together with our data, were analyzed using the neighbor-joining method with 1000 bootstrap replicates and the genetic distances were estimated using the Kimura-2-parameter. At the morphological level, the Brazilian population cannot be distinguished either from *Chironomus striatipennis* or *Chironomus kiiensis*, configuring a species complex but, at the molecular level our studied population is placed in a clade together with *Chironomus striatipennis*, from South Korea. Bionomic characteristics of the Brazilian *Chironomus* population differ from the ones of *Chironomus kiiensis* from Japan, the only species in this species complex with bionomic information available. The Brazilian *Chironomus* population has a smaller size, the double of the number of eggs and inhabits oligotrophic water, in artificial container. In the molecular analysis, populations of *Chironomus striatipennis* and *Chironomus kiiensis* are placed in a clade, formed by two groups: Group A (which includes populations from both named species, from different Asiatic regions and our Brazilian population) and Group B (with populations of *Chironomus kiiensis* from Japan and South Korea). Genetic distance between the Brazilian population and specimens in Group A suggests that it was recently introduced in Brazil, and that its country of origin is probably South Korea.

## Introduction

Alien species represent one of the most serious threats to biodiversity at different taxonomic levels (Mack et al. 2000) including freshwater ecosystems (Gherardi 2007). Human activities have been contributing to the increase and to the strengthening of this process (Lodge 1993) as many species can be transported around the world on human transportation systems such as ships, airplanes and automobiles. Examples include the green crab (*Carcinus maenas* Linnaeus, 1758), mud crab (*Rhithropanopeus harrisii* Gould, 1841) and blue mussel (*Mytilus galloprovincialis* Lamark, 1819), which were recorded being transported in ballast tanks (Briski et al. 2012). The Asian tiger mosquito (*Aedes albopictus* (Skuse, 1894)), which is a vector of the dengue viruses, was introduced in several countries through the importation of tires from Asia (Fontenille and Toto 2001).

Despite their dominance in terms of diversity in most freshwater ecosystems, aquatic insects have a disproportionately low number of alien species when compared to other freshwater macroinvertebrates (Karatayev et al. 2009). Exceptions include several examples of recognized alien species of Ephemeroptera (Zimmermann 1957), including one in Brazil, a Baetidae species from Africa reported in Brazil’s Espirito Santo state (Salles et al. 2014).

Among the necessary characteristics for a species to become a successful invasive alien are: phenotypic plasticity, ability for uniparental reproduction and fast growth in disturbed habitats (Kleunen et al. 2010). Additional important characteristics for alien aquatic insects are: generalist feeding (e.g., detritivores), year-round breeding capacity, ability to colonize peri-urban environments and artificial water bodies, and the climatic similarity of invaded and source environments (De Moor 1992). Chironomidae species often have characteristics mentioned above, and cases of successfully introduced *Chironomus* species have been reported around the world (Jacobsen and Perry 2007, Hribar et al. 2008; Gray et al. 2012).

During a study on aquatic insects in Amazonas state (Brazil), we collected specimens of Chironomidae that were similar, at the morphological level, to *Chironomus kiiensis* Tokunaga and, we have named it as such (Lacerda et al. 2014). However, Martin (2014) reported that *Chironomus striatipennis* Kieffer is morphologically similar to *Chironomus kiiensis* and, that the latter is treated as a junior synonym of *Chironomus striatipennis*; cytogenetic studies also indicated that both species are included in the pseudothummi-cytocomplex species. Both species have geographic distribution restricted to Asia. *Chironomus striatipennis* is widely distributed in South and Southeast Asia and it is a common species in rice fields and other wetlands in India (Chaudhuri and Chattopadhyay 1990), while *Chironomus kiiensis* is reported as the most prevalent species in South Korea and Japan (Ree 1993). The problem is that identification of these species is based, mainly, on their geographical distribution since it is not possible to distinguish them at the morphological level (Martin 2014). This fact results in specimens collected in South and Southeast Asia being identified as *Chironomus striatipennis* and those collected in South Korea and Japan being identified as *Chironomus kiiensis* (e.g., Nath and Lakhotia 1989; Yong et al. 1999; Jeong et al. 2004; Nandi et al. 2011).

In view of this complex situation, our objectives were to register a *Chironomus* population of this Asiatic species complex in Brazil, to provide morphological information on this population, to investigate its identity using DNA barcoding and, to provide bionomic information about this species.

## Methods

### Study area and field collection

Egg masses of the *Chironomus* Brazilian population were collected in tap water accumulated in a 10 L plastic container, for several days (5^th^, 6^th^, 8^th^, 10^th^ and 14^th^), in January 2011 in the urban area of Manaus municipality Amazonas, Brazil (03°06'50.17"S, 59°58'30.99"W). The egg masses were placed individually in 80 mL plastic vials, which were labeled with collection information, covered with a screen and observed daily until the larvae hatched and abandoned the gelatinous mass. Larvae from each egg batch were transferred to a plastic tray (19.5×31×6.5 cm) containing burned sand as substrate and 1.5 L of water (pH = 5.9; electrical conductivity of 20.7 µS cm^-1^). The trays were covered with wooden structures (40×21×32 cm) serving as frames for screens (2 mm mesh), following a model modified from Fonseca and Rocha (2004) for retention of adults and behavioral observations.

Larvae were fed fish food (TETRAMIM®) every 48 hours. The colony established using this collected material was kept in the insect-raising facility at the Coordenação de Biodiversidade, Instituto Nacional de Pesquisas da Amazônia (INPA), at environmental conditions similar to those of the climate in Manaus during the months from May to September 2011: temperature of 26 ± 0.3 °C; air humidity of 75 ± 7.7% and photoperiod of 12/12 hours (data obtained from http://www.inmet.gov.br). The specimens analyzed in the present study were obtained from this colony.

### Species identification based on morphological and molecular analysis

Emerged adults with pupal and larval exuviae from the colony were dissected and mounted on slides with Euparal® following the procedures outlined by Epler (1988). The morphological characteristics analyzed were male genitalia, anal spur and distribution of shagreen in pupae and, in structures present on the larval head capsule (antenna, pectin epipharyngis, premandible, mandible, mentum, labral setae). Comparisons between the Brazilian *Chironomus* population (n = 40; 10 adult male, 10 adult female, 10 pupae and 10 larvae) and other *Chironomus* species were made using available species descriptions and related literature (Kieffer 1910; Tokunaga 1936; Chaudhuri et al. 1992; Martin 2014). We also examined specimens of *Chironomus striatipennis* (n = 4; 1 male adult, 1 pupa and 2 larvae) from India (lent by Dr. Jon Martin, University of Melbourne, Australia) and specimens of *Chironomus kiiensis* (n = 30; 10 male adult, 5 adult female, 5 pupae and 10 larvae) from Japan (lent by Dr. Masaru Yamamoto, from Yamaguchi Prefecture). The measurements and images presented in this study were obtained using an Olympus compound microscope with a mounted digital photographic camera, model Olympus DP72, using Cell D (Olympus) software and a stereomicroscope (Leica M165C), with Leica software (auto montage, Application Suite V3).

Molecular analyses were done using the DNeasy Blood & Tissue (Qiagen) kit following the manufacturer’s recommendations. Amplifications of the extracted DNA from three specimens (2 larvae and 1 adult male) of the Brazilian *Chironomus* population were made using primers developed by Folmer et al. (1994) that are specific for the Cytochrome Oxidase I (COI) gene in the mitochondrial DNA. The amplified fragments were purified by the EXO-SAP (Exonuclease I-Shrimp Alkaline Phosphatase) method and sent to the Centro de Estudo do Genoma Humano (Universidade de São Paulo), where they were sequenced using an ABI 3730 DNA Analyzer.

Sequences for 14 *Chironomus* species and *Lipiniella fujiprimus* (Sasa, 1985) available at the GenBank and Barcode of Life Data Systems (BOLD) were used in the analysis (accession numbers in Table 1). The alignment and editing of the sequences were done in BioEdit software v.5.0.6 (Hall 1999). A tree was constructed using the neighbor-joining method with 1000 bootstrap replicates, and genetic distances were estimated using the Kimura-2-parameter (K_2_P) model in the Mega5 program (Tamura et al. 2011).

Voucher specimens of the Brazilian population are deposited in the Coleção de Invertebrados do Instituto Nacional de Pesquisas da Amazônia. Haplotype sequences are deposited in GenBank under the accession numbers KJ424334–KJ424336.

**Table 1.** GenBank and BOLD accession numbers of the sequences from species of *Chironomus* and *Lipiniella fujiprimus* included in the analysis.

Species	Accession numbers	Reference
*Chironomus kiiensis*	JF412086–JF412089*; KC407765*; AB838642–AB838646*; JQ350720*; AB740240–AB740241*	Kim et al. 2012 -
*Chironomus striatipennis*	COTW008-08, COTW011-08, COTW012-08, COTW027-10**	-
*Chironomus balatonicus*	JN016827*	-
*Chironomus calligraphus*	KF278357*; COTW041-11–COTW042-11**	Proulx et al. 2013 -
*Chironomus columbiensis*	COTW001-08–COTW002-08**	-
*Chironomus curabilis*	JN016822*	-
*Chironomus flaviplumus*	JF412077*	Kim et al. 2012
*Chironomus javanus*	JF412085*	Kim et al. 2012
*Chironomus nipponensis*	JN887053*	-
*Chironomus plumosus*	JF412198*	Kim et al. 2012
*Chironomus salinarius*	KC250756*	Kim et al. 2012
*Chironomus usenicus*	JN016819*	-
*Chironomus xanthus*	DQ648209*	-
*Lipiniella fujiprimus*	JF412078*	Kim et al. 2012

*GenBank; **BOLD; - data unpublished.

### Biological information

The egg masses were characterized by their shape, length, width and number of eggs; the maximum length and width of eggs were measured. In order to determine the development time of the egg stage, five egg masses were isolated and observed every hour until first instar hatched.

Ten egg masses were isolated to determine the development time of each of the four larval instars; starting from the moment at which the first instar hatched, three larvae were fixed (in 80% ethanol) daily, until the last larva of the egg mass pupated. To identify the instar of each of the fixed larvae; they were mounted between slide and coverslip (using Hoyer as the mounting medium) to measure the ventral length of the head capsule, following the methodology of Strixino (1973). To classify larvae into one of the four larval instars, the measurements obtained were subjected to a frequency-distribution analysis; each peak in the graph indicates a larval instar (Strixino 1973). The development time of each instar was determined based on the size limits of each larval instar obtained in the frequency-distribution graph and the day when they were preserved. The development time of each larval instar was determined by combining information gathered from the frequency distribution graph with the measurements of the head capsule of the larvae collected daily.

To estimate pupal development time, 50 pupae were observed every 12 hours from the moment of pupation until adult emergence. Longevity of adults was estimated using 50 adults that emerged in the laboratory on the same day; these were isolated in pairs (male and female) in cages made of PET bottles and observed until there were no more survivors.

## Results

### Morphological analysis

**Figure 1.**
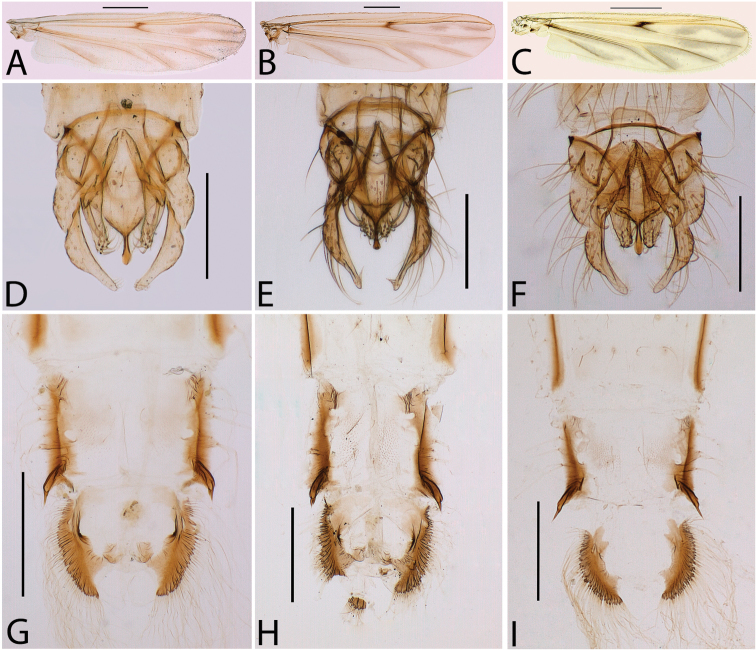
Adult male and pupae. *Chironomus striatipennis*, Indian population. **A** Wing **D** Hypopygium, dorsal view **G** Anal spur, dorsal view. *Chironomus kiiensis*, Japanese population **B** Wing **E** Hypopygium, dorsal view **H** Anal spur, dorsal view. *Chironomus striatipennis*, Brazilian population **C** Wing **F** Hypopygium, dorsal view **I** Anal spur, dorsal view. Scale bar: 500 µm (**A, B, C, G, H, I**); 200 µm (**D, E, F**).

The morphology of the Brazilian *Chironomus* population (adult, pupal and larval) is identical to that presented in the original descriptions of *Chironomus striatipennis* and of *Chironomus kiiensis* (Kieffer 1910; Tokunaga 1936), and in other taxonomic papers on these two species (Chaudhuri et al. 1992; Martin 2014), it being impossible to distinguish them from each other morphologically. The examined specimens of *Chironomus striatipennis* (Fig. 1A, D, G) from India and of *Chironomus kiiensis* (Fig. 1B, E, H) from Japan were also morphologically indistinguishable from the Brazilian *Chironomus* population (Fig. 1C, F, I).

### Molecular analysis

**Figure 2.**
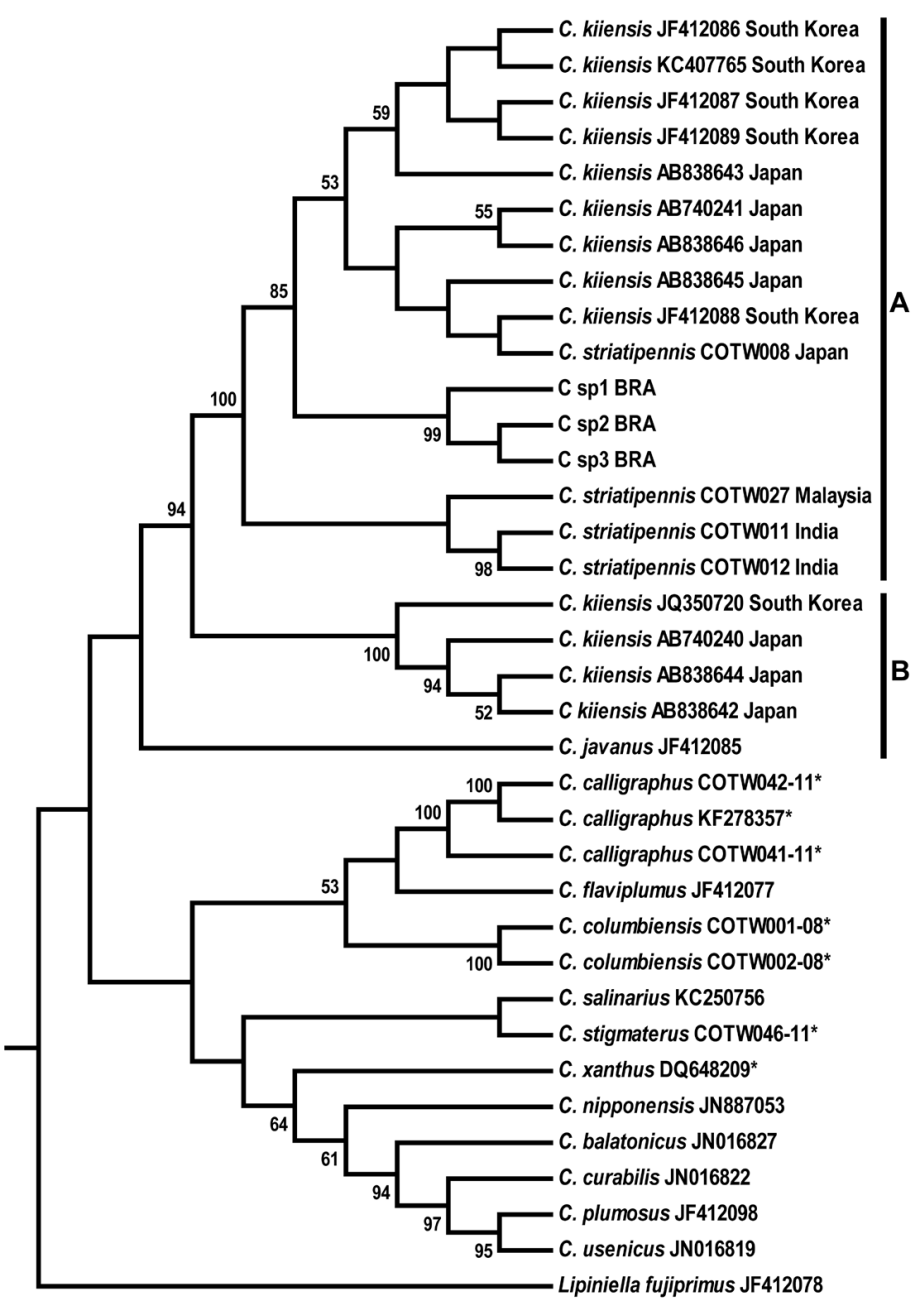
NJ tree based on the COI sequences of the mtDNA of *Chironomus* (Diptera: Chironomidae) species. The sequence of *Lipiniella fujiprimus* was used as the outgroup. Bootstrap values > 50% are shown on branches. Accession numbers and countries are provided beside the species names. Species flagged with an asterisk (*) are neotropical species. Brazilian *Chironomus* population: *Chironomus* sp1BRA; *Chironomus* sp2BRA; *Chironomus* sp3BRA

In the neighbor-joining tree (Fig. 2), we observed that the Brazilian *Chironomus* population (*Chironomus* sp1BRA, *Chironomus* sp2BRA and *Chironomus* sp3BRA) grouped, with 94% bootstrap support, with others specimens of *Chironomus striatipennis* and of *Chironomus kiiensis* from different regions of East Asia. The analysis resulted in two groups, each with 100% bootstrap support, which we named “Group A” and “Group B”. Group A is composed of the Brazilian *Chironomus* population and specimens identified as *Chironomus kiiensis* from South Korea and Japan and *Chironomus striatipennis* from Malaysia and India. Group B included specimens identified as *Chironomus kiiensis*, also from South Korea and Japan.

*Chironomus* species from other regions, including the three specimens from Brazil, were included in a distinct clade (Fig. 2). Considering that Groups A and B represent monophyletic groups, based on the intraspecific genetic distance between their members, we observe that in Group A the genetic distance varied from 0.0 to 3.3%, while in Group B the distance varied from 0.6 to 2.0% (Table 2). The average genetic distance between sequences from Groups A and B was 9.6% (Table 3), and within each group the average genetic distance was 1.3%.

**Table 2.** Genetic distance between Groups A and B and other *Chironomus* species based on the COI gene in the mtDNA. Values in bold in the first two lines indicate genetic distance within each group, and in the remaining lines bold values indicate intraspecific genetic distances. Analyses were conducted using Kimura-2-parameter model.

		1	2	3	4	5	6	7	8	9	10	11	12	13
1.	Group A	**1.3**												
2.	Group B	9.6	**1.3**											
3.	*Chironomus nipponensis*	17.0	15.6											
4.	*Chironomus plumosus*	17.2	19.5	12.0										
5.	*Chironomus flaviplumus*	14.4	13.3	13.5	17.9									
6.	*Chironomus javanus*	13.3	13.6	15.4	16.7	15.0								
7.	*Chironomus curabilis*	17.2	17.0	11.0	5.5	16.3	17.4							
8.	*Chironomus usenicus*	16.5	17.5	10.5	3.1	16.8	17.2	4.0						
9.	*Chironomus balatonicus*	18.2	17.2	12.0	8.8	16.1	16.5	8.4	8.2					
10.	*Chironomus salinarius*	17.9	15.7	15.4	18.7	15.1	18.3	16.4	17.0	18.4				
11.	*Chironomus calligraphus*	14.8	16.1	16.1	17.2	12.1	15.5	17.2	16.2	16.3	17.0	**1.8**		
12.	*Chironomus columbiensis*	13.7	12.2	14.3	15.7	11.7	13.8	15.6	15.0	16.6	16.6	12.3	**1.2**	
13.	*Chironomus stigmateus*	16.9	15.6	16.1	18.6	16.2	15.3	17.0	17.6	19.6	15.7	17.5	15.7	
14.	*Chironomus xanthus*	10.5	10.6	10.1	5.9	13.8	9.4	7.4	6.7	6.5	12.3	9.7	10.5	13.8

**Table 3.** Pairwise per cent nucleotide differences (p-distance) between all specimens based on the COI gene in the mtDNA. Analyses were conducted using the Kimura-2-parameter model. Values in bold are genetic distances between Groups A and B.

		1	2	3	4	5	6	7	8	9	10	11	12	13	14	15	16	17	18	19
1.	*Chironomus* sp1BRA																			
2.	*Chironomus* sp2BRA	0.0																		
3.	*Chironomus* sp3BRA	0.0	0.0																	
4.	*Chironomus kiiensis*_JF412086_South_Korea	0.8	0.8	0.8																
5.	*Chironomus kiiensis*_JF412087_South_Korea	0.8	0.8	0.8	0.0															
6.	*Chironomus kiiensis*_JF412089_South_Korea	0.8	0.8	0.8	0.0	0.0														
7.	*Chironomus kiiensis*_KC407765_South_Korea	0.8	0.8	0.8	0.0	0.0	0.0													
8.	*Chironomus kiiensis*_JF412088_South_Korea	1.1	1.1	1.1	0.3	0.3	0.3	0.3												
9.	*Chironomus kiiensis*_AB740241_Japan	1.2	1.2	1.2	0.6	0.6	0.6	0.6	0.6											
10.	*Chironomus kiiensis*_AB838643_Japan	1.7	1.7	1.7	0.9	0.9	0.9	0.9	1.2	1.5										
11.	*Chironomus kiiensis*_AB838645_Japan	0.9	0.9	0.9	0.2	0.2	0.2	0.2	0.2	0.5	1.1									
12.	*Chironomus kiiensis*_AB838646_Japan	0.9	0.9	0.9	0.3	0.3	0.3	0.3	0.3	0.3	1.2	0.2								
13.	*Chironomus striatipennis*_COTW008_Japan	0.9	0.9	0.9	0.2	0.2	0.2	0.2	0.2	0.5	1.1	0.0	0.2							
14.	*Chironomus striatipennis*_COTW011_India	3.0	3.0	3.0	2.5	2.5	2.5	2.5	2.8	3.1	3.3	2.6	2.8	2.6						
15.	*Chironomus striatipennis*_COTW012_India	2.8	2.8	2.8	2.6	2.6	2.6	2.6	2.6	2.9	3.3	2.4	2.6	2.4	1.3					
16.	*Chironomus striatipennis*_COTW027_Malaysia	2.8	2.8	2.8	2.2	2.2	2.2	2.2	2.2	1.9	3.1	2.0	1.9	2.0	3.3	2.8				
17.	*Chironomus kiiensis*_AB740240_Japan	**9.2**	**9.2**	**9.2**	**9.1**	**9.1**	**9.1**	**9.1**	**9.2**	**9.2**	**9.7**	**9.1**	**9.2**	**9.1**	**10.4**	**10.0**	**9.6**			
18.	*Chironomus kiiensis*_AB838642_Japan	**9.1**	**9.1**	**9.1**	**9.2**	**9.2**	**9.2**	**9.2**	**9.4**	**9.4**	**9.9**	**9.2**	**9.4**	**9.2**	**10.3**	**9.8**	**9.9**	0.6		
19.	*Chironomus kiiensis*_AB838644_Japan	**9.2**	**9.2**	**9.2**	**9.4**	**9.4**	**9.4**	**9.4**	**9.6**	**9.6**	**10.3**	**9.4**	**9.6**	**9.4**	**10.1**	**9.6**	**9.9**	0.6	0.6	
20.	*Chironomus kiiensis*_JQ350720_South_Korea	**10.1**	**10.1**	**10.1**	**10.1**	**10.1**	**10.1**	**10.1**	**10.3**	**9.9**	**11.2**	**10.1**	**9.9**	**10.1**	**11.1**	**10.7**	**10.3**	1.9	2.2	2.0

Within Group A there are three specimens of *Chironomus striatipennis* from Malaysia and India with a mean genetic divergence of 2.6%, a higher value considering that the mean divergence between the remaining group members was 0.6% (Table 3). The mean genetic distance between Groups A, B and other *Chironomus* species, including Neotropical species, was 15.6%, ranging from 10.5 to 19.5% (Table 3).

### Biological information

**Figure 3.**
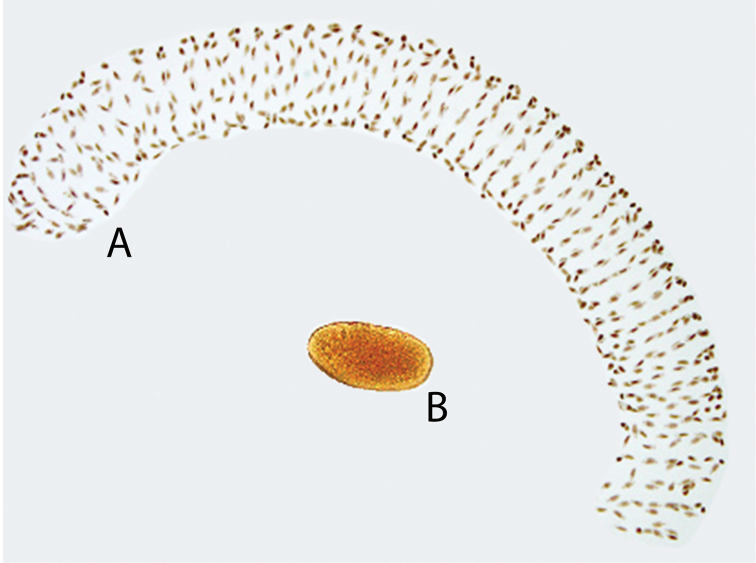
*Chironomus striatipennis*, Brazilian population. **A** Egg mass **B** Egg.

Egg masses of the Brazilian *Chironomus* population were found attached by a stem in the wall of a plastic tray with water. The egg masses measured 16.6 mm (SD = 3.1; n = 5) in length and 1.59 mm (SD = 0.04; n = 5) in width (median region). Each mass contained an average of 600 eggs (SD = 104; n = 5). The eggs were elliptical in shape and measured, on average, 0.24 mm (SD = 0.02; n = 50) in length and 0.10 mm (SD = 0.01; n = 50) in width. The mean incubation time of the eggs was two days (SD = 0.5; n = 50) at 26 °C, with a hatching rate of 89.9%. The eggs were distributed in a pseudo-spiral pattern, in alternate parallel rows along the primary axis of the gelatinous mass (Fig. 3A) and eggs were eliptic format (Fig. 3B).

**Figure 4.**
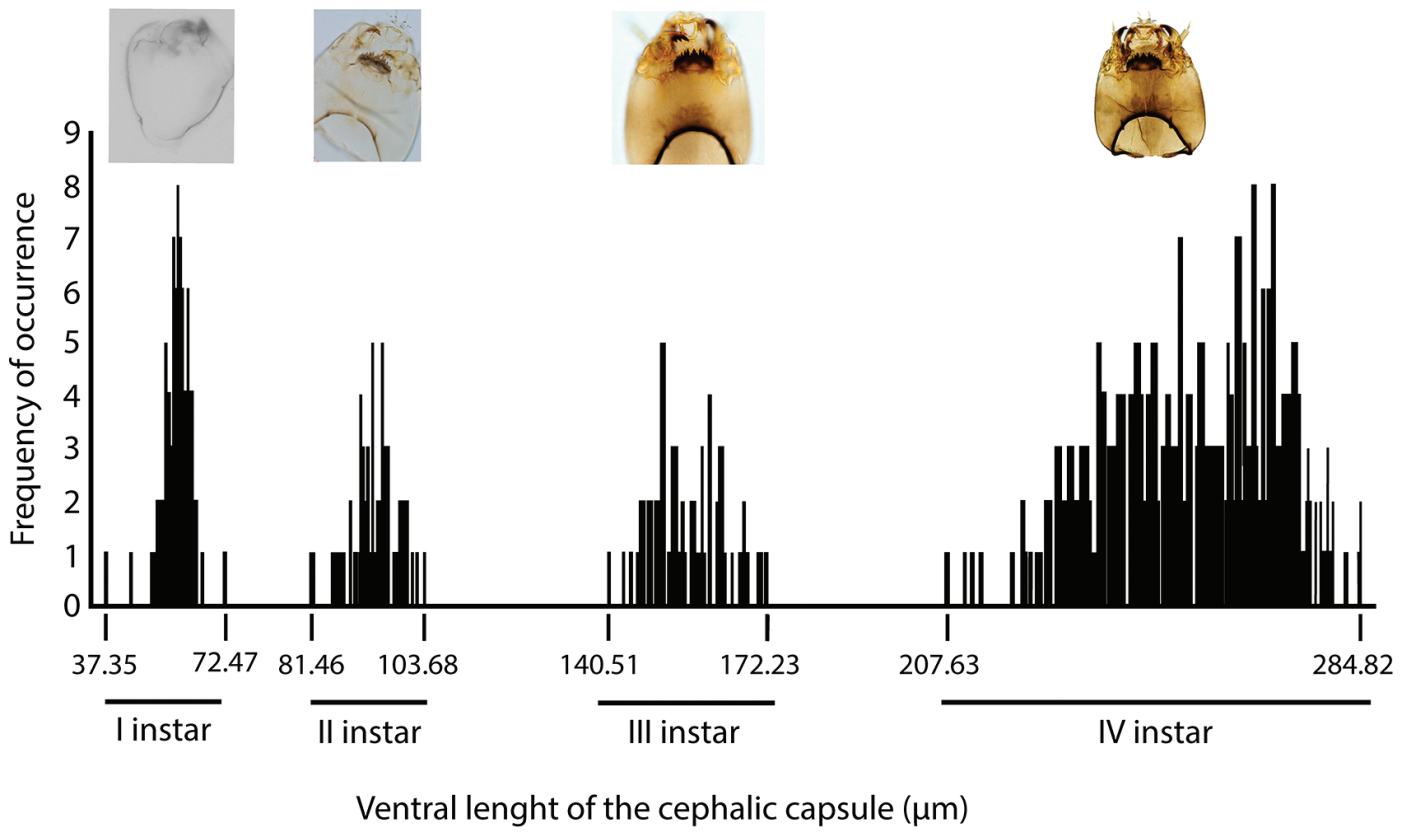
Frequency of occurrence of the ventral length of the cephalic capsule of a Brazilian *Chironomus* population (Diptera: Chironomidae) showing the four larval instars.

The four instars of the Brazilian *Chironomus* population were well defined using the ventral length of the head capsule. Mean length of the head capsule of the 1^st^-instar is 54.7 µm (SD = 4.2; n = 81); the 2^nd^ 93.7 µm (SD = 4.5; n = 53); the 3rd 157.1 µm (SD = 7.2; n = 67) and the 4^th^ 257.6 µm (SD = 16.7; n = 316) (Fig. 4).

Development times of the 1^st^ (SD = 0.6; n = 81), 2^nd^ (SD = 1.0; n = 53) and 3^rd^ instars (SD = 1.1; n = 67) were similar, each averaging three days; the 4^th^ instar was the longest, with mean development time of 10 days (SD = 2.5; n = 316). Mean time for complete larval stage development was 19 days (SD = 5.2; n = 517). Mean development time for the pupal stage, at 26 °C, was two days (SD = 0.24, n = 50). The life span of adults, on average, at 26 °C, was three days for both males (SD = 0.70, n = 50) and females (SD = 0.65, n = 50). Development time of the Brazilian *Chironomus* population from the time larvae hatch to the adult stage was 27 days at 26°C. The emergence percentage was 42.9% for females and 57.1% for males.

## Discussion

### Species identification

High morphological similarities observed between the Brazilian *Chironomus* population and both *Chironomus striatipennis* and *Chironomus kiiensis* (Kieffer 1910; Tokunaga 1936) corroborate the studies that reported the difficulty in distinguishing the latter two species at the morphological level, including the one that proposed *Chironomus kiiensis* as a synonym of *Chironomus striatipennis* (Martin 2014). However, cytotaxonomic studies have indicated that they are sibling species that belong to the same cytocomplex (Martin 2014). Our molecular analysis showed that both species names have been applied to specimens with high genetic divergence, and that the identification based on geographical distribution, which is currently in common usage to circumscribe both species (e.g., Nath and Lakhotia 1989; Yong et al. 1999; Jeong et al. 2004; Nandi et al. 2011), is not a good taxonomic practice.

Studies on the genetic intraspecific divergence in some Chironomidae genera have reported values between 0.5 and 2.3% (Sinclair and Gresens 2008; Silva et al. 2013; Proulx et al. 2013; Trivinho-Strixino et al. 2012). Similar values were observed in studies on other insects, including some Diptera families (e.g., Beckenbach and Borkent 2003; Hamada et al. 2010; Hernández-Triana et al. 2012). Although Silva et al. (2013) attributed low intraspecific divergence (mean 0.91%) to the specimens having been collected in the same place, our results and other studies, for example, with Lepidoptera and Simuliidae, do not corroborate this hypothesis (e.g., Hebert et al. 2004; Hernández-Triana et al. 2012).

The interspecific genetic divergence observed in our data is in accordance with results for other groups of Chironomidae and other aquatic Diptera families, with values around 15% (Beckenbach and Borkent 2003; Sinclair and Gresens 2008; Proulx et al. 2013; Silva et al. 2013), corroborating the hypothesis that Groups A and B represents at least two distinct species. Group A is composed of specimens identified as *Chironomus striatipennis* and *Chironomus kiiensis* from different regions of Asia. Within this group, there are three specimens identified as *Chironomus striatipennis* from Malaysia and India (the country where this species was described) with high genetic divergence (mean 2.62%) when compared to other members in Group A (Table 3.). This fact might be an indication of the presence of two sibling species in Group A. But, since we have no detailed information on the morphology of all life stages of the populations included in Group A, we suggest that this group should be treated as a species complex. Based on ICNZ rules (Principle of Priority – Article 23.3), and as encouraged by Martin (2014), we named Group A as the *Chironomus striatipennis* species complex.

The large genetic distance (mean 9%) between Group B specimens (composed of specimens identified as *Chironomus kiiensis* from Japan and South Korea) and Group A is a clear indication that each clade represents a distinct species. This is also corroborated by the genetic distance observed between specimens in each species group (maximum = 3.3%), and by the interspecific genetic distance values reported by other studies. For example, a study on three *Podonomus* populations observed that the genetic distances between them were greater than 7%, indicating the presence of three species (Trivinho-Strixino et al. 2012). Since *Chironomus kiiensis* was described from Japan, we hypothesized that the specimens in Group B may represent this species. On the other hand, because of the lack of information on the morphology of all life stages of these populations, and based on genetic information obtained from GenBank for specimens collected near the type locality of *Chironomus kiiensis*, we choose do not propose any name for Group B specimens.

The placement of the Brazilian *Chironomus* population in Group A and its small genetic distance from South Korean specimens (0.8%) indicates that this might represents a recently introduced species in Brazil, probably, from a population from South Korea. This could have occurred due to the fact that Manaus is located in a port zone, receiving many cargo ships from different continents, including Asia, due to the presence of industries in the Manaus Tax-Free Zone (SUFRAMA). We therefore assume that specimens of the Brazilian *Chironomus* species arrived in Brazil by ship. Other exotic species have been entering the country by this way, perhaps using ballast water or some other source of standing water (e.g. Santos and Lamonica 2008; Brodin and Anderson 2009; Raunio et al. 2009; Jensen 2010).

### Biological information

Environmental conditions where the Brazilian *Chironomus* population and the Japanese *Chironomus kiiensis* population were collected were different: the Brazilian population was observed inhabiting oligotrophic water and the Japonese population eutrophic water (Inoue et al. 2008; Al-Shami et al. 2010). The Brazilian *Chironomus* population has double the number of eggs (~ 600 eggs) compared to *Chironomus kiiensis* from Japan, although they were reared at similar water temperatures (25–26 °C), feeding methodology and food type were not the same (Maeda and Yano 1988). Larval development time of the Brazilian *Chironomus* population was shorter (10 days) than that of the Japanese *Chironomus kiiensis* population (14–20 days) (Nandi et al. 2011). Development times of the 1^st^, 2^nd^ and the 3^rd^ instars of the Brazilian *Chironomus* population was shorter than that of the 4^th^ instar, as has also been observed in other *Chironomus* species (Fonseca and Rocha 2004; Zilli et al. 2008). The 4^th^ instar’s long larval development time is probably due to the greatest body growth occurring in this period (Tokeshi 1995), as well the production and maturation of the oocytes (Strixino and Trivinho-Strixino 1985).

The size of last-instar larvae, represented by the ventral length of the head capsule, of the Brazilian *Chironomus* population was smaller than that of the Japanese *Chironomus kiiensis* population (Maeda and Yano 1998). Male survival time of the Brazilian *Chironomus* population under laboratory conditions was shorter (three days) than in the Japanese *Chironomus kiiensis* population at similar air temperature (25 °C) (Maeda and Yano 1988; Nandi et al. 2011). Environmental conditions can have an effect on species biology and information in this area is important for characterizing the habitat of each species in a species complex and can help to delimit each species in the complex.

### Concluding remarks

In this study we addressed the taxonomic problem involving the species named *Chironomus striatipennis* and *Chironomus kiiensis* from Asia, since these names have been used for specimens morphologically similar and closely related. Regional variation among Asian populations of the two above mentioned species was observed, but only at molecular level, based on the genetic distance estimated using a partial COI sequence. We hypothesize that these two names encompass at least two, and perhaps three, species, based on sequences deposited in GenBank and BOLD. We indicated that the usual way to identify these two species, which are practically indistinguishable at the morphological level, based on geographical distribution is not a feasible approach, since specimens identified with the same name (*Chironomus kiiensis*) from Japan and South Korea have highly genetic divergent (genetic distance of 9%). A detailed study, including the morphology of all life stages and a multilocus molecular analysis of populations from the entire distribution needs to be done to solve this taxonomic problem. The presence of an Asiatic *Chironomus* in Brazil might be the consequence of human globalization, where fast and easy global transportation systems are available to any organisms with the minimum characteristics of alien species. This example also demonstrates that the geographic limits of species cannot be considered in isolation, but rather need to be examined from a broad perspective to avoid mistakes.
